# Effects of Fish Oil, Lipid Mediators, Derived from Docosahexaenoic Acid, and Their Co-Treatment against Lipid Metabolism Dysfunction and Inflammation in HFD Mice and HepG2 Cells

**DOI:** 10.3390/nu15020427

**Published:** 2023-01-13

**Authors:** Yan Su, Hack-Sun Choi, Jong-Hyun Choi, Hee-Sik Kim, Gil-Yong Lee, Hee-Won Cho, Heonsik Choi, Yong-Suk Jang, Jeong-Woo Seo

**Affiliations:** 1 Microbial Biotechnology Research Center, Korea Research Institute of Bioscience and Biotechnology (KRIBB), Jeongeup-si 56212, Republic of Korea; 2 Department of Bioactive Material Sciences, The Institute for Molecular Biology and Genetics, Jeonbuk National University, Jeonju 54896, Republic of Korea; 3 Faculty of Biotechnology, College of Applied Life Sciences, Jeju National University, Jeonju 63243, Republic of Korea; 4 Cell Factory Research Center, Korea Research Institute of Bioscience and Biotechnology (KRIBB), Daejeon-si 34141, Republic of Korea; 5 Healthcare Technology Institute, Kolon Advanced Research Center, 110 Magokdong-ro, Seoul 07793, Republic of Korea

**Keywords:** nonalcoholic fatty liver disease, inflammation, lipid metabolism, fish oil, lipid mediators

## Abstract

Although fish oil (FO) and lipid mediators (LM) derived from polyunsaturated fatty acids can prevent obesity, their combined effects and cellular metabolism remain unclear. Therefore, this study aimed to examine the potential protective and metabolic effects of FO in combination with LM (a mixture of 17S-monohydroxy docosahexaenoic acid, resolvin D5, and protectin DX [3:47:50], derived from docosahexaenoic acid (DHA)) on palmitic acid (PA)-induced HepG2 cells and high-fat- diet (HFD)-induced C57BL/6J mice after 9-week treatment. Lipid metabolism disorders and inflammation induced by HFD and PA were substantially reduced after FO and LM treatment. Further, FO and LM treatments reduced lipid accumulation by increasing fatty acid oxidation via peroxisome proliferator-activated receptor α and carnitine-palmitoyl transferase 1 as well as by decreasing fatty acid synthesis via sterol regulatory element-binding protein-1c and fatty acid synthase. Finally, FO and LM treatment reduced inflammation by blocking the NF-κB signaling pathway. Importantly, the combination of FO and LM exhibited more robust efficacy against nonalcoholic fatty liver disease, suggesting that FO supplemented with LM is a beneficial dietary strategy for treating this disease.

## 1. Introduction

Nonalcoholic fatty liver disease (NAFLD) is a major risk factor for human health. It is associated with obesity, insulin resistance, Type 2 diabetes, and metabolic syndrome [1,2,3]. Several clinical drugs for NAFLD have been recalled because of their adverse effects, such as decreased appetite and cardiotoxicity, whereas others have not provided the expected benefits [4,5]. Several studies are aiming to identify efficient and safe treatment strategies for NAFLD [6].

NAFLD is a metabolic syndrome that is triggered by an imbalance in fatty acid synthesis and oxidation [7,8]. Peroxisome proliferator-activated receptors (PPAR) are crucial for controlling the gene expression of the various enzymes involved in fatty acid oxidation, including catalase, acyl-CoA oxidase 1, and carnitine-palmitoyl transferase 1 (CPT-1) [9]. In contrast, sterol regulatory element-binding protein-1 (SREBP-1) stimulates the expression of genes associated with lipogenesis, including acetyl-CoA carboxylase and fatty acid synthase (FAS) [10,11].

Obesity is associated with the chronic activation of inflammatory pathways [12]. Several recent reports have demonstrated that the NF-kB signaling pathway plays a key role in the liver, adipose tissue, and central nervous system in the development of HFD disease, inducing in subsequent inflammation through the high secretion of proinflammatory cytokines, such as tumor necrosis factor alpha (TNF-α), interleukin-1 beta (IL-1β), and interleukin-6 (IL-6) [8,9,13,14].

A range of studies have demonstrated that fish oil (FO), rich in *n*-3 PUFAs, has beneficial effects in terms of NAFLD [15,16,17,18]. FO alleviates lipid imbalance, reduces oxidative stress indicators, and restores liver function in dyslipidemic rats, slowing the development of NAFLD [16,17]. The *n*-3 PUFAs is the main effective content in fish oil to contribute to anti-inflammatory properties, regulating metabolic and inhibiting NAFLD, including eicosapentaenoic acid (EPA; 20:5, *n*-3), docosahexaenoic acid (DHA; 22:6, *n*-3), and docosapentaenoic acid (DPA; 22:5, *n*-3) [19,20]. Despite the benefits of FO, increasing evidence has indicated that specialized proresolving lipid mediators (SPMs) derived from EPA and DHA (known as maresins, resolvins, and protectins) exert greater anti-inflammatory effects at approximately 1000 times lower doses; on the other hand, diet with DPA could increase the level of SPM in specific tissue and blood, and thus also contribute to the effect of SPM [20,21]. Resolvin D1 or maresin 1 administered intraperitoneally at a dose of 2 μg/kg improved glucose tolerance, reduced adipose tissue dysfunction and inflammation, and increased the phosphorylation levels of AKT or AMPK in adipose tissues in obese diabetic mice [22,23].

Several studies have focused on combined approaches for NAFLD, providing improved benefits through similar or different pathways [24,25]. Although fish oil and lipid mediators present antiobesity effects, robust combination effects have not been observed on NAFLD. This study aims to assess the potential effects of fish oil supplement with lipid mediators on lipid metabolic profiles and inflammation both in vivo and in vitro.

## 2. Materials and Methods

### 2.1. Materials

FO (EPA C20:5 *n*-3: 60 mg/g, DHA, C22:6, *n*-3: 460 mg/g, DPA, C22:5 *n*-3 + *n*-6, 105 mg/g) was acquired from KD Nutra (Branttvag, Norway). A mixture of lipid mediators (LM) was obtained from DHA through enzymatic reaction using soybean lipoxygenase, which is composed of 17S-monohydroxy DHA, resolvin D5, and protectin DX at ratio of 3:47:50. Palmitic acid (PA) was purchased from Fluka (Seelze, Germany). Human liver cancer cell line HepG2 was purchased from Korea Cell Line Bank (Seoul, Republic of Korea). A bicinchoninic acid (BCA) assay kit was purchased from Sango Company (San Diego, CA, USA). Antibodies against IKKB (ab32135), *p*-IKKB (ab194528), IKB (ab32518), *p*-IKB (ab133462), NF-kB (p65) (ab16502), *p*-NF-kB (*p*-p65) (ab76302), and GAPDH (ab181602) were purchased from Abcam (Cambridge, MA, USA). Enhanced chemiluminescence (ECL) was purchased from Bio-Rad Laboratories, Inc. (Tewksbury, MA, USA). The assay kits for triacylglycerol (TG), total cholesterol (TC), high-density lipoprotein (HDL) cholesterol, and low-density lipoprotein (LDL) cholesterol were purchased from BioVision (Milpitas, CA, USA). Mouse/human IL-6 and TNF-α ELISA kits were purchased from Abcam (Cambridge, MA, USA).

### 2.2. Animals and Treatments

All animal experiments were governed according to the hosting facility’s guidelines for animal handling and welfare, as described by the Institutional Animal Care and the Use Committee of the Korea Research Institute of Bioscience & Biotechnology (Daejeon, Republic of Korea), and they were approved by the Institutional Animal Ethics Committee (KRIBB-AEC-21216) prior to initiation of the study. Male C57BL/6J (5 weeks old, 17–19 g) mice were obtained from Orient bio (Gyeonggi, Republic of Korea) and were maintained under regular environmental conditions (21–23 °C and 60–70% relative humidity) with a 12 h light/dark cycle. After acclimatization for 1 week, the mice were randomly separated into five groups (*n* = 8/group): (1) NC, (2) HFD, (3) HFD + FO, (4) HFD + LM, and (5) HFD + LM + FO. LM and FO were orally administered daily at dose of 10 μg/kg body weight and 250 mg/kg body weight, respectively, whereas NC and HFD groups were administered an equal volume of sterile saline via gavage for 9 weeks. The LM was diluted into 2 μg/mL with saline, FO was diluted into 5% (*w*/*w*) with olive oil, and the mice were orally administered daily at 5 μL/g body weight. Dosages chosen for the present study were based on preliminary-dose-ranging studies with FO and LM in vivo and in vitro. The mice in the NC group were fed a normal chow diet (LabDiet, 5053, 11.3% calories from fat), whereas mice in other groups were fed a HFD (Research Diets, D12492, 60% calories from fat). During treatment, body weight was measured once a week.

### 2.3. Biochemical Parameters Assay

After 9 weeks of treatment, the mice were euthanized after a 12 h fast. Blood was then collected and centrifuged (8000 rpm, 4 °C, 10 min) for the preparation of serum. The levels of TG, TC, HDL, LDL, IL-6, and TNF-α in serum were determined according to the manufacturer’s instructions. Similarly, liver tissue was collected after 9 weeks of treatment, and the TG level in hepatic homogenate was determined in accordance with the manufacturer’s instructions.

### 2.4. Histological Analysis

Liver and epididymal adipose tissues from the studies above were embedded in paraffin after being fixed in a 10% formalin solution. Hematoxylin and eosin (H&E) were used to stain paraffin-embedded tissue slices (5 μm) [26]. A microscope (Leica, Bensheim, Germany) was employed to investigate all stained sections.

### 2.5. Cell Culture and Treatment

HepG2 cells were cultured in Dulbecco’s Modified Eagle Medium (DMEM) enriched with 10% FBS and 100 IU/mL penicillin and 100 μg/mL streptomycin (Gibico, Thermo Fisher Scientific, San Jose, CA, USA) at 37 °C and 5% CO_2_. The PA solution was produced as previously described, albeit with a small change [27]. After being dissolved in 1 M NaOH and heated to 70 °C, 0.5% bovine serum albumin (BSA; Sigma-Aldrich, St. Louis, MO, USA) was immediately mixed with PA and diluted in DMEM. The HepG2 cells were divided into five groups: (1) NC, where the cells were grown in DMEM with 0.5% BSA supplement as control group; (2) PA, where the cells were incubated with 150 μM PA solution promoting lipid accumulation; (3) PA + FO, where the cells were pretreated with FO at 1 mg/mL for 6 h followed by further stimulation with PA at 150 μM for 18 h; (4) PA + LM, where the cells were pretreated with LM at 1 μg/mL for 6 h and then further stimulated with PA at 150 μM for 18 h; and (5) PA + LM + FO, where the cells were treated with FO combined with LM for 6 h followed by incubation with PA till 24 h.

### 2.6. TG Assay in HepG2 Cells

HepG2 cells were pretreated with FO, LM, or LM plus FO for 6 h and then further incubated in 150 μM PA for 18 h. Cells were collected, and the TG level assay was performed according to the manufacturer’s instructions.

### 2.7. qRT-PCR

Total RNA was isolated using the TaKaRa MiniBEST kit (TaKaRa, Tokyo, Japan) according to the manufacturer’s instructions. Transcript levels were measured using a One-Step AccuPower GreenStar RT-qPCR PreMix kit using SYBR Green, according to the manufacturer’s instructions (Bioneer Corporation, Daejeon, Republic of Korea). Reverse transcription polymerase chain reaction (RT-PCR) was performed in a reaction volume of 50 μL. Quantitative real-time PCR was performed in a CFX Connect system (Bio-Rad, Hercules, CA, USA). GAPDH was used as the housekeeping gene. The relative mRNA expression levels of the target genes were determined using the 2^−ΔΔCT^ method. Table 1 presents all specific primers.

### 2.8. Western Blotting

The protein was collected from the liver tissue homogenates and cells using RIPA buffer (Biosolution, Seoul, Republic of Korea) with 1:100 protein protease inhibitor cocktail, 1:100 PMSF, and 1:100 phosphatase inhibitor for 30 min on ice. The samples were then centrifuged at 12,000 rpm for 5 min at 4 °C, and the protein concentration was determined using BCA kit and heated with a 4× loading buffer (Solarbio, Beijing, China) for 10 min at 100 °C. Equal protein quantities (~25 μg) were then separated in 7.5% and 10% SDS-polyacrylamide gels and transferred on 0.45 μm PVDF membranes (Millipore, Bedford, MA, USA) at 20 V for 120 min. Membranes were then blocked with Tris-buffered saline/Tween 20 (TBST) containing 5% skim milk for 45 min and were incubated with primary antibodies against NF-kB (p65) (1:20,000), *p*-NF-kB (pp65) (1:1000), IKB (1:5000), *p*-IKB (1:10,000), IKKB (1:5000), *p*-IKKB (1:1000), and GAPDH (1:10,000) at 4 °C for overnight incubations. Membranes were washed with TBST (×3) and incubated with appropriate horseradish peroxidase (HRP)-conjugated secondary antibodies (1:50,000, Abcam, Cambridge, MA, USA) at room temperature for 2 h. The membranes were then incubated with Clarity Western ECL Substrate (Bio-Rad, Hercules, CA, USA) after being washed with TBST and finally exposed to CL-XPosure film (Thermo Scientific, Rockford, IL, USA). The gray density of the target protein bands was quantified using ImageJ software (National Institutes of Health, Maryland, MD, USA).

### 2.9. Statistical Analysis

Data were expressed as means ± SDs. Statistical analysis of the results was performed by one-way ANOVA using GraphPad prism 7.0 (GraphPad, San Diego, CA, USA). The results were considered statistically significant for *p* < 0.05.

## 3. Results

### 3.1. Diet with FO and LM Reduced the Body Weight and Adipose Tissue of HFD Mice

**Figure 1:**
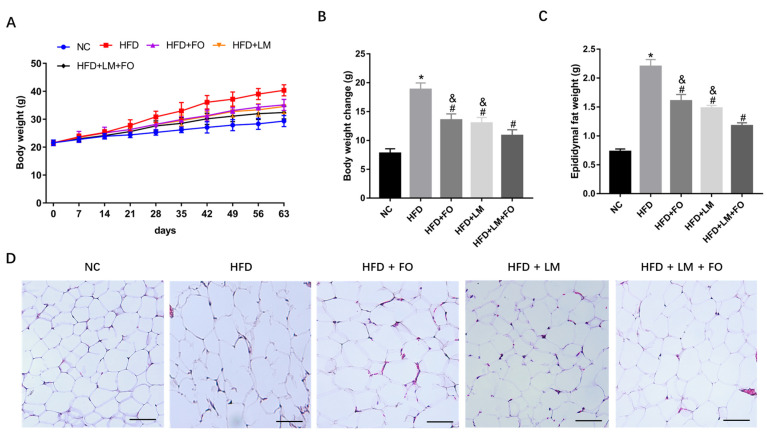
Effects of FO and LM on body weight and adipose tissue in HFD mice. Male C57BL/6J mice were randomly grouped into NC (sterile saline), HFD (sterile saline), HFD + FO (FO at 250 mg/kg), HFD + LM (LM at 10 μg/kg), and HFD + LM + FO (FO at 250 mg/kg and LM at 10 μg/kg), and orally administrated daily for 9 weeks. (**A**) Body weight was recorded per week during the experiments. (**B**) Body weight gain, (**C**) adipose weight, and (**D**) H&E staining of epididymal adipose tissue (200×, scar bar = 100 μm) were measured after the 9-week treatment period. Data are presented as means ± SD (*n* = 8). * *p* < 0.05 vs. NC; ^#^ *p* < 0.05 vs. HFD; ^&^ *p* < 0.05 vs. LM + FO.

HFD mice were stimulated by being fed with HFD containing 60% fat for 9 weeks. As shown in Figure 1A,B, the body weight was significantly increased in the HFD group relative to the NC group. Nevertheless, FO alone, LM alone, and FO supplemented with LM treatments all displayed significant decreases in body weight gain. In addition, treatment with FO plus LM showed synergic effects relative to FO or LM alone on the reduction of body weight.

As shown in Figure 1C,D, the epididymal adipose tissue was greater in HFD mice than in NC mice, along with greater adipocytes presented in H&E staining. The epididymal adipose tissues and adipocytes were notably reduced after all treatments. Taken together, all of the above results suggest that FO or LM alone and, in particular, FO supplemented with LM, played a key role against obesity in HFD mice.

### 3.2. Diet with FO and LM Reversed Serum Lipid Profile in HFD-Fed Mice

**Figure 2:**
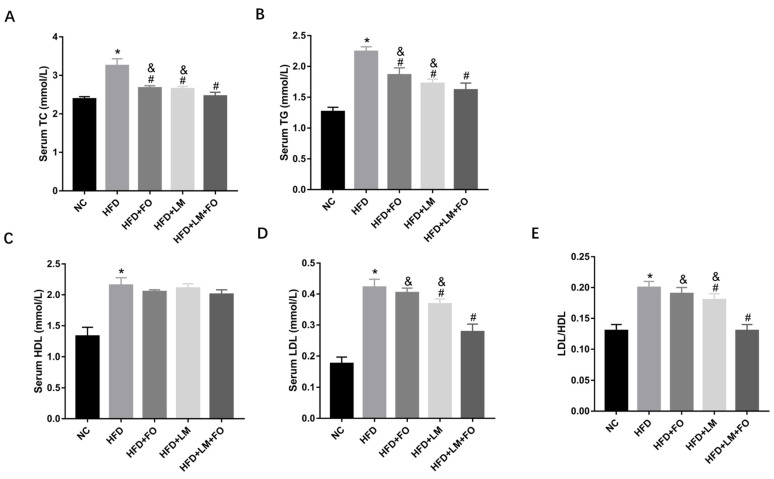
Effects of FO and LM on serum lipid accumulation in HFD mice. Serum lipid profile involved (**A**) TC, (**B**) TG, (**C**) HDL, and (**D**) LDL, and (**E**) the ratio of LDL/HDL was detected by kits. Data are presented as means ± SD (*n* = 3). * *p* < 0.05 vs. NC; ^#^ *p* < 0.05 vs. HFD; ^&^ *p* < 0.05 vs. LM + FO.

After a 9-week treatment period, the levels of TC, TG, HDL, and LDL in serum were determined. As shown in Figure 2A–E, all parameters significantly increased in the HFD group, demonstrating that the high-fat diet induced hyperlipidemia. The FO diet alone significantly decreased the accumulation of TG and TC relative to HFD mice, whereas no significant difference was observed in terms of HDL, LDL, and LDL/HDL. On the other hand, LM alone significantly reduced the levels of TC, TG, LDL, and LDL/HDL ratio. FO supplemented with LM, as expected, presented the optimal effect on suppressing the HFD-induced increase of TC, TG, LDL, and LDL/HDL ratio. These findings indicated that serum lipid accumulation was significantly reduced by FO alone, LM, and FO combined with LM, whereas the combination of FO and LM showed greater effects than FO or LM alone.

### 3.3. Diet with FO and LM Recovered Fatty Liver in HFD-Fed Mice

**Figure 3:**
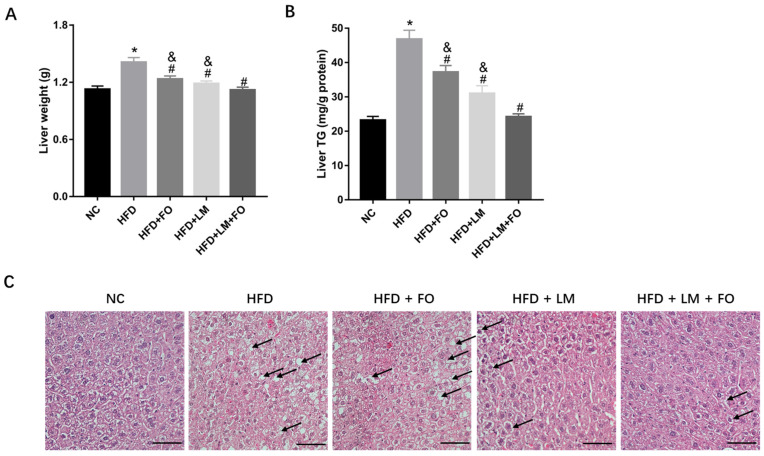
Effects of FO and LM on liver injury in HFD mice. (**A**) Liver weight determined after 9 weeks of treatment. (**B**) TG levels in liver determined as described in the text. (**C**) Histological analysis of liver sections evaluated after hematoxylin and eosin (H&E) staining (400×, scar bar = 50 μm). Black arrows indicated steatosis. Data are presented as means ± SD (*n* = 3). * *p* < 0.05 vs. NC; ^#^ *p* < 0.05 vs. HFD; ^&^ *p* < 0.05 vs. LM + FO.

Compared to NC mice, HFD resulted in a larger liver, which was notably decreased after treatment with FO or LM alone or FO supplemented with LM (Figure 3A). Accordingly, the accumulation of TG in the liver was significantly higher in HFD liver, whereas it was then significantly decreased following the three different treatments (Figure 3B). In addition, H&E staining showed that the liver in the HFD group exhibited a high degree of steatosis, which was reversed following treatment with FO alone, LM alone, and especially FO supplemented with LM (Figure 3C). Taken together, the combination with FO and LM produced a stronger effect on recovering fatty liver caused by HFD.

### 3.4. Diet with FO and LM Improved Lipid Metabolism Dysfunction in HFD-Fed Mice

**Figure 4:**
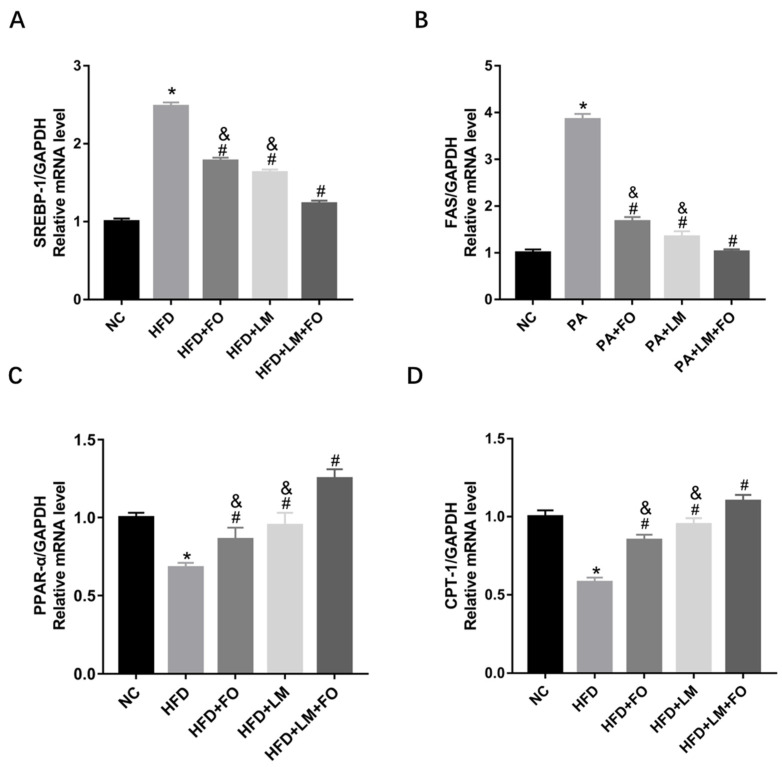
The effects of FO and LM on lipid metabolism in HFD mice. Real-time PCR was employed to determine mRNA expression of adipogenesis genes in the liver, including (**A**) SREBP-1, (**B**) FAS, (**C**) PPARα, and (**D**) CPT-1. Data are presented as means ± SD (*n* = 3). * *p* < 0.05 vs. NC; ^#^ *p* < 0.05 vs. HFD; ^&^ *p* < 0.05 vs. LM + FO.

The liver is a key organ for the metabolism of lipids; the genes that stimulated TG synthesis and are implicated in lipogenesis and fatty acid β-oxidation were examined [28,29]. The mRNA expression of SREBP-1 and FAS, which are correlated with fat acid synthesis, were significantly increased in the liver of HFD mice. However, SREBP-1 and FAS were significantly decreased after treatment with FO or LM, or in particular FO supplemented with LM (Figure 4A,B). PPARα and CPT-1, associated with the fatty acid β-oxidation, were clearly reduced after feeding with HFD. As expected, gene expression was significantly increased by FO or LM, especially FO supplemented with LM (Figure 4C,D). Thus, the data suggest that FO and LM regulated lipid metabolism in the liver, and the impact was stronger when FO and LM were combined.

### 3.5. Diet with FO and LM Ameliorated Liver Inflammation in HFD-Fed Mice

**Figure 5:**
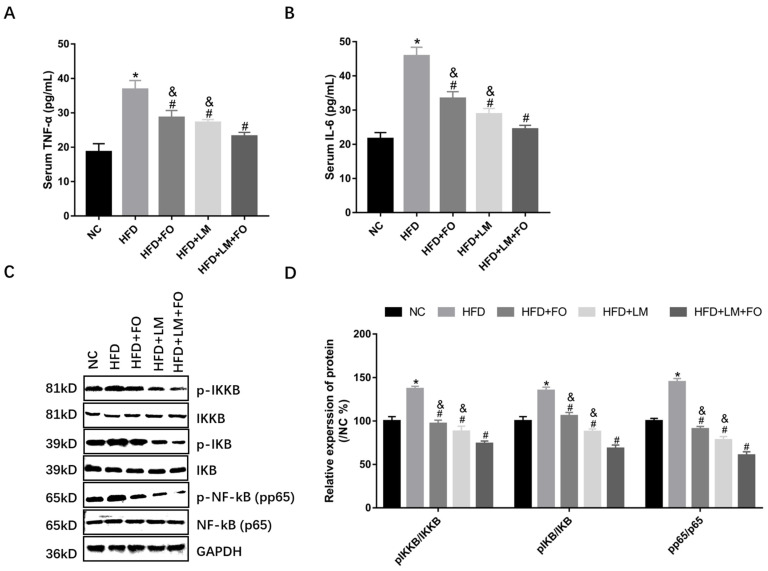
Effects of FO and LM on liver inflammation in HFD mice. The levels of (**A**) TNF-α and (**B**) IL-6 in serum were determined using ELISA, as described in the text. (**C**) Protein was detected by Western blot for the proteins of interest, including *p*-IKKB, IKKB, *p*-IKB, IKB, *p*-NF-kB (pp65), and NF-kB (p65). GAPDH was used as a reference protein. (**D**) Relative expression of *p*-IKKB/IKKB, *p*-IKB/IKB, and *p*-NF-kB (pp65)/NF-kB (p65) were calculated by ImageJ. Data are presented as means ± SD (*n* = 3). * *p* < 0.05 vs. NC; ^#^ *p* < 0.05 vs. HFD; ^&^ *p* < 0.05 vs. LM + FO.

Inflammation is an essential factor in the development of NAFLD [12]. The levels of serum TNF-α and IL-6, which are associated with inflammation, were significantly higher in HFD mice than in NC mice (Figure 5A,B). Interestingly, treatment with FO alone, LM alone, and FO in combination with LM effectively decreased the levels of TNF-α and IL-6 (Figure 5A,B).

The expression levels of *p*-IKKB, *p*-IKB, and *p*-NF-KB (pp65) were then determined by Western blotting, undertaken to investigate the molecular mechanisms of the anti-inflammatory effects on NAFLD. As shown in Figure 5C,D, the expression of *p*-IKKB/IKKB, *p*-IKB/IKB, and *p*-NF-KB (pp65)/NF-kB (p65) were markedly upregulated in HFD mice. By contrast, the expression of these phosphorylated proteins was significantly downregulated following treatment with FO alone, LM alone, or FO supplemented with LM. Moreover, FO plus LM resulted in greater declines of proinflammatory cytokines and the NF-kB signaling pathway. Taken together, these indicated that FO alone, LM alone, and FO supplemented with LM inhibited liver inflammation through the NF-kB signaling pathway.

### 3.6. FO and LM Controlled PA-Induced Lipid Accumulation and Lipid Metabolism in HepG2 Cells

**Figure 6:**
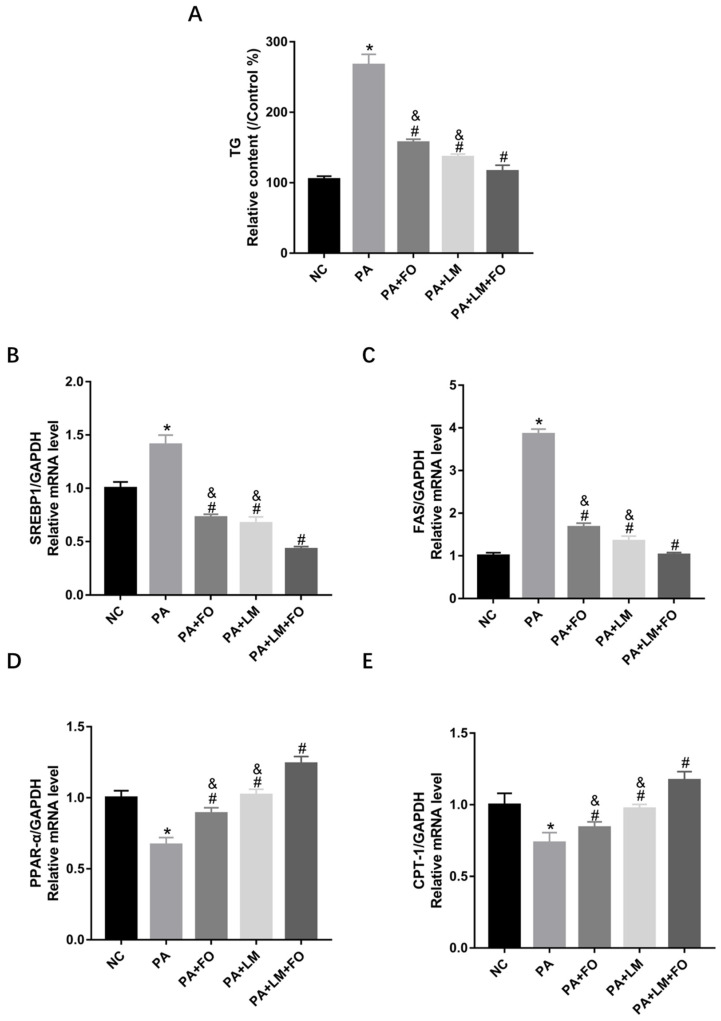
Effects of FO and LM on PA-induced lipid accumulation and lipid metabolism in HepG2 cells. (**A**) Lipid accumulation was evaluated by assessing the TG levels. Real-time PCR was used to detect the mRNA expression of adipogenesis genes in HepG2 cells, including (**B**) SREBP-1, (**C**) FAS, (**D**) PPARα, and (**E**) CPT-1. Data are presented as means ± SD (*n* = 3). * *p* < 0.05 vs. NC; ^#^ *p* < 0.05 vs. PA; ^&^ *p* < 0.05 vs. LM + FO.

To validate the effects of FO alone, LM alone, and FO supplemented with LM with respect to lipid accumulation, we performed in vitro studies using HepG2 cells. As shown in Figure 6A, TG level was significantly increased after incubation with 150 μM PA; however, it was reduced after incubation with FO alone, LM alone, and FO supplemented with LM.

Furthermore, gene expression related to the lipid metabolism was detected. As shown in Figure 6B–E, SREBP-1 and FAS were significantly upregulated, whereas PPARα and CPT-1 were notably downregulated after incubation with FO supplemented with LM compared with PA group, FO alone, and LM alone. These findings suggested that compared to FO or LM alone, FO supplemented with LM presented a more robust effect in reducing the lipid accumulation by controlling lipid synthesis and fatty acid β-oxidation.

### 3.7. FO and LM Altered PA-Induced Inflammation in HepG2 Cells

**Figure 7:**
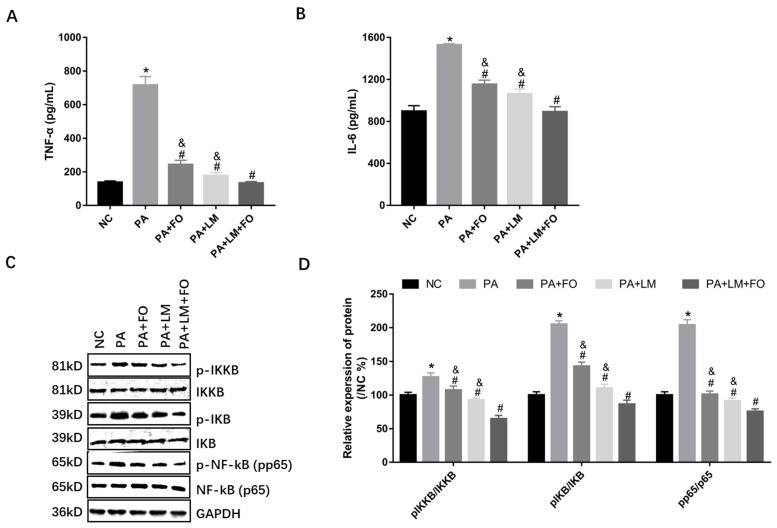
Effects of FO and LM on PA-induced inflammation in HepG2 cells. Levels of (**A**) TNF-α and (**B**) IL-6 in cell supernatants were detected. (**C**) Protein expression was detected by Western blot, including *p*-IKKB, IKKB, *p*-IKB, IKB, *p*-NF-kB (pp65), and NF-kB (p65). GAPDH was used as a reference protein. (**D**) Relative expression of *p*-IKKB/IKKB, *p*-IKB/IKB, and *p*-NF-kB (pp65)/NF-kB (p65) were calculated by ImageJ. Data are presented as means ± SD (*n* = 3). * *p* < 0.05 vs. NC; ^#^ *p* < 0.05 vs. PA; ^&^ *p* < 0.05 vs. LM + FO.

Levels of TNF-α and IL-6 were significantly increased after stimulation with 150 μM PA, and they were then greatly inhibited by incubation with FO alone, LM alone, or FO supplemented with LM (Figure 7A,B). Moreover, the expression of *p*-IKKB/IKKB, *p*-IKB/IKB, and *p*-NF-kB (pp65)/NF-kB (p65) were significantly higher in PA-treated HepG2 cells, whereas the expression of these phosphorylation proteins was reversed by incubation with either FO alone, LM alone, or FO supplemented with LM (Figure 7C,D). In addition, FO combined with LM seemed to be more effective against inflammation. Thus, these observations indicated that FO alone, LM alone, and FO supplemented with LM inhibited inflammation by blocking NF-kB signaling pathways.

## 4. Discussion

The incidence of NAFLD is increasing, owing to the increasing rates of unhealthy habits; among these, HFD is considered to be a key risk factor [30]. Nevertheless, there are few effective drugs in clinical trials, which indicates the need to pursue safe compounds as alternative therapies for treating NAFLD [2,4]. Obesity is usually accompanied by insulin resistance, lipid accumulation, and liver dysfunction, and, in some cases, by liver steatosis following chronic inflammation and oxidative stress [5,31,32]. In this study, the effects of FO or LM alone and FO supplemented with LM against dyslipidemia, lipid metabolism disorders, and inflammation were investigated, both in vivo (C57/BL 6J mice) and in vitro (HepG2 cells). Moreover, FO and LM reversed lipid accumulation in blood and liver by promoting lipid oxidation (PPARα, CPT-1), as well as inhibiting lipid synthesis (SREBP, FAS). In addition, excessive lipid resulted in chronic inflammation, along with higher secretion of proinflammatory cytokines, such as TNF-α and IL-6. All treatments significantly reduced liver inflammation associated with the inhibition of NF-kB signaling pathway.

In this study, it was highlighted that FO and LM not only decreased body weight and adipose tissue, but also significantly regulated the lipid profile in serum. Lipid accumulation, which can be stored in lipid droplets or secreted into the blood, is the main characteristic of hepatic steatosis [10]. Moreover, high-fat diet increased the levels of TC, TG, LDL, and HDL in serum. We found that FO only decreased the levels of TC and TG, without influence on LDL or HDL. However, LM or FO combined with LM decreased the levels of TC, TG, and LDL, and had no effect on HLD. Furthermore, LM or FO plus LM decreased the ratio of LDL/HDL, which is known to be a standard tool to evaluate cardiovascular risk [33]. Consistently, we also observed that FO and LM decreased TG levels in HepG2 cells when compared to PA-treated cells. These results suggested that FO combined with LM was a potential therapy for NAFLD.

In NAFLD, hepatic fat accumulation results from an imbalance between lipid acquisition and lipid disposal mediated, related increased lipogenesis, and insufficient fatty acid oxidation [34,35]. We observed abnormal TG accumulation in liver after long-term high-fat diet, which was supported by the high degree of steatosis examined by H&E. Furthermore, FO and LM declined the TG level and alleviated the steatosis in liver. PPARα, a member of the PPAR family, is essential for the regulation of fatty acid β-oxidation through the stimulation of associated protein expression, such as CPT-1 [9]. FAS is an enzyme for fatty acid synthesis, and its metabolism and homeostasis are induced by SREBP-1 in response to feeding habits and insulin resistance [10]. As a result, this study discovered that FO and LM significantly upregulated the expression of PPARα and CPT-1, whereas downregulated the expression of FAS and SREBP-1 both in vivo and in vitro, which resulted in the reduction of TG level [34]. Likewise, it was demonstrated that FO and SPM reduced lipid accumulation in blood and liver by an induction of β-oxidation of fatty acids and an inhibition of fatty acid synthesis [16,25]. 

HFD commonly promotes inflammation, which is characterized by the elevated secretion of proinflammatory cytokines (TNF-α, IL-6, and IL-1β), which is associated with the NF-kB signaling pathway [12,36,37]. It has been previously reported that PD1 and 17-hydroxy-DHA improve insulin sensitivity and repress TNF-α, IL-6, and monocyte chemotactic protein 1 (MCP-1) genes in HFD-fed mice or ob/db mice [36]. Similarly, Resolvin D1 (2 μg/kg) decreased adipose tissue macrophage accumulation and IL-6 in obese diabetic mice [22]. DHA, hydroxytyrosol, and its metabolites resolved WAT inflammation through downregulation of the NF-kB system [25]. In the present study, we also observed that high-fat diet caused chronic inflammation, characterized by the increase of TNF-α and IL-6, along with the activation of the NF-kB signaling pathway. Furthermore, FO and LM blocked the NF-kB signaling pathway and decreased the proinflammation cytokines in vivo and in vitro.

Some studies revealed that lipid metabolism may correlate with inflammation [26,37,38]. It was demonstrated that downregulation of the expression of PPARα promotes inflammation [38]. Our study also suggested that FO and LM downregulated the expression of the NF-kB and upregulated the expression of PPARα, showing that the expressions of NF-kB and PPAR-α were negatively correlated. Thus, we speculate that the molecular interaction between NF-kB and PPARα in liver may be potentially therapeutic targets for the treatment of hepatic diseases.

Many kinds of studies indicate that fish oil and their lipid mediators present antiobesity effects; robust combination effects have not been observed on NAFLD. We discovered that FO and LM produced a stronger effect on lipid metabolic profiles and inflammation in NAFLD. Moreover, both the FO and the LM were mixtures of potentially bioactive compounds, so further research will be required to establish which ingredients are the most bioactive. However, the current data mainly focused on the basic parameters to reveal the effects of FO and LM on inflammation and lipid metabolism in HFD and HepG2 cells. Prior to clinical implementation, more research is still required to demonstrate the underlying mechanisms, such as what triggers inflammation, the mechanism of FO and LM on stimulating lipid metabolism (PI3K/AKT and AMPK signaling pathway), the interaction of lipid metabolism and inflammation, and the effects on insulin resistance.

## 5. Conclusions

From the evidence presented above, it is suggested that FO and LM can reduce lipid accumulation, reverse lipid metabolism disorders, and inhibit inflammation both in vivo and in vitro. FO and LM decrease fatty acid synthesis and improve fatty acid oxidation. In addition, all treatments prevent liver inflammation by inactivating the NF-kB pathway. Moreover, FO supplemented with LM forms a more effective therapy for reversing the serum lipid dysfunction, lipid metabolism, and inflammation. In conclusion, a diet with FO supplemented with LM could be an efficient adjuvant therapy for patients with obesity and liver damage.

## Figures and Tables

**Table 1:** Primer sequences.

Genes	Sequences (5′ → 3′)
Human PPAR-α	Forward: TCGGCGAGGATAGTTCTGGAAG
	Reverse: GACCACAGGATAAGTCACCGAG
Human CPT-1	Forward: GATCCTGGACAATACCTCGGAG
	Reverse: CTCCACAGCATCAAGAGACTGC
Human SREBP-1	Forward: ACTTCTGGAGGCATCGCAAGCA
	Reverse: AGGTTCCAGAGGAGGCTACAAG
Human FAS	Forward: TTCTACGGCTCCACGCTCTTCC
	Reverse: GAAGAGTCTTCGTCAGCCAGGA
Human GAPDH	Forward: GTCTCCTCTGACTTCAACAGCG
	Reverse: ACCACCCTGTTGCTGTAGCCAA
Mouse PPAR-α	Forward: TCATCAAGAAGACCGAGTCC
	Reverse: CCTCTTCATCCCCAAGCGT
Mouse CPT-1	Forward: CATCCACGCCATACTGCT
	Reverse: GACCTTGAAGTAACGGCCTC
Mouse SREBP-1	Forward: GGCACTAAGTGCCCTCAACCT
	Reverse: GCCACATAGATCTCTGCCAGTGT
Mouse FAS	Forward: GGCACCTATGGCGAGGACTT
	Reverse: GCCCTCCCGTACACTCACTC
Mouse GAPDH	Forward: CATCACTGCCACCCAGAAGACTG
	Reverse: ATGCCAGTGAGCTTCCCGTTCAG
Human PPAR-α	Forward: TCGGCGAGGATAGTTCTGGAAG
	Reverse: GACCACAGGATAAGTCACCGAG
Human CPT-1	Forward: GATCCTGGACAATACCTCGGAG
	Reverse: CTCCACAGCATCAAGAGACTGC
Human SREBP-1	Forward: ACTTCTGGAGGCATCGCAAGCA
	Reverse: AGGTTCCAGAGGAGGCTACAAG
Human FAS	Forward: TTCTACGGCTCCACGCTCTTCC
	Reverse: GAAGAGTCTTCGTCAGCCAGGA
Human GAPDH	Forward: GTCTCCTCTGACTTCAACAGCG
	Reverse: ACCACCCTGTTGCTGTAGCCAA

## Data Availability

Not applicable.
